# Overactive Bladder Symptoms Within Nervous System: A Focus on Etiology

**DOI:** 10.3389/fphys.2021.747144

**Published:** 2021-12-10

**Authors:** Chuying Qin, Yinhuai Wang, Yunliang Gao

**Affiliations:** Department of Urology, The Second Xiangya Hospital, Central South University, Changsha, China

**Keywords:** overactive bladder, nerve system disease, detrusor overactivity, lower urinary tract symptoms, etiology

## Abstract

Overactive bladder (OAB) is a common debilitating condition characterized by urgency symptoms with detrimental effects on the quality of life and survival. The exact etiology of OAB is still enigmatic, and none of therapeutic approaches seems curative. OAB is generally regarded as a separate syndrome, whereas in clinic, OAB symptoms could be found in numerous diseases of other non-urogenital systems, particularly nervous system. The OAB symptoms in neurological diseases are often poorly recognized and inadequately treated. This review provided a comprehensive overview of recent findings related to the neurogenic OAB symptoms. Relevant neurological diseases could be mainly divided into seven kinds as follows: multiple sclerosis and related neuroinflammatory disorders, Parkinson’s diseases, multiple system atrophy, spinal cord injury, dementia, peripheral neuropathy, and others. Concurrently, we also summarized the hypothetical reasonings and available animal models to elucidate the underlying mechanism of neurogenic OAB symptoms. This review highlighted the close association between OAB symptoms and neurological diseases and expanded the current knowledge of pathophysiological basis of OAB. This may increase the awareness of urological complaints in neurological disorders and inspire robust therapies with better outcomes.

## Introduction

Overactive bladder (OAB) was defined as a storage symptom syndrome characterized by “urinary urgency, usually accompanied by frequency and nocturia, with or without urgency urinary incontinence, in the absence of urinary tract infection or other obvious pathology” (Abrams et al., 2002). The prevalence of OAB increases with advancing age and is greatly varied across studies. A population-based survey included over 19,000 participants and demonstrated an overall prevalence of OAB to be 11.8% (10.8% in men and 12.8% in women). Other studies have reported the prevalence of up to 30–40% (Coyne et al., 2009). Despite great strides made in the past decades, the exact etiology of OAB is still enigmatic and none of therapeutic approaches seems curative. As OAB is a separate syndrome, its symptoms could also be found in numerous diseases of other non-urogenital systems, such as diabetes, cardiovascular diseases, and sleep disorders. Given the basis of the condition relying on a subjective symptom of urgency, available animal models with indirect or surrogate markers of urgency have been applied for basic science research into OAB. Therefore, exploration of the highly observed comorbidity between OAB symptoms and other diseases could potentially shed light on the pathophysiology of OAB and address the confusing situation hampering research and management.

An accumulating evidence has demonstrated that OAB symptoms are regarded as significant features in numerous neurological diseases, such as multiple sclerosis (MS), spinal cord injury (SCI), Parkinson’s disease (PD), stroke, and spina bifida (Panicker et al., 2015; Yamada et al., 2018). The OAB symptoms are highly prevalent among neurogenic patients, as shown by a recent study that over 50% of these patients reported OAB symptoms (Przydacz et al., 2021). This can be largely anticipated from the crucial regulatory effect of nervous system on the micturition reflex. Also, the severity of OAB symptoms varies with the type and degree of damage to the nervous system. However, the OAB symptoms in neurological diseases are often poorly recognized and relatively few of individuals with these symptoms seek care (Dmochowski and Newman, 2007; Przydacz et al., 2021). Moreover, since OAB symptoms in neurogenic patients often have their own particularities, respected clinical efficacy may not be achieved by referring to the conventional treatment programs of OAB. Even worse, older adults with neurocognitive dysfunction are at higher risk of taking multiple medications with anticholinergic properties for OAB symptoms (Duong et al., 2021). As yet, up to now only a scarcity of studies could totally present OAB symptoms in these neurological diseases and clearly summarize the possible mechanisms underlying this tight connection (Chapple et al., 2017; Cornu, 2017).

**FIGURE 1:**
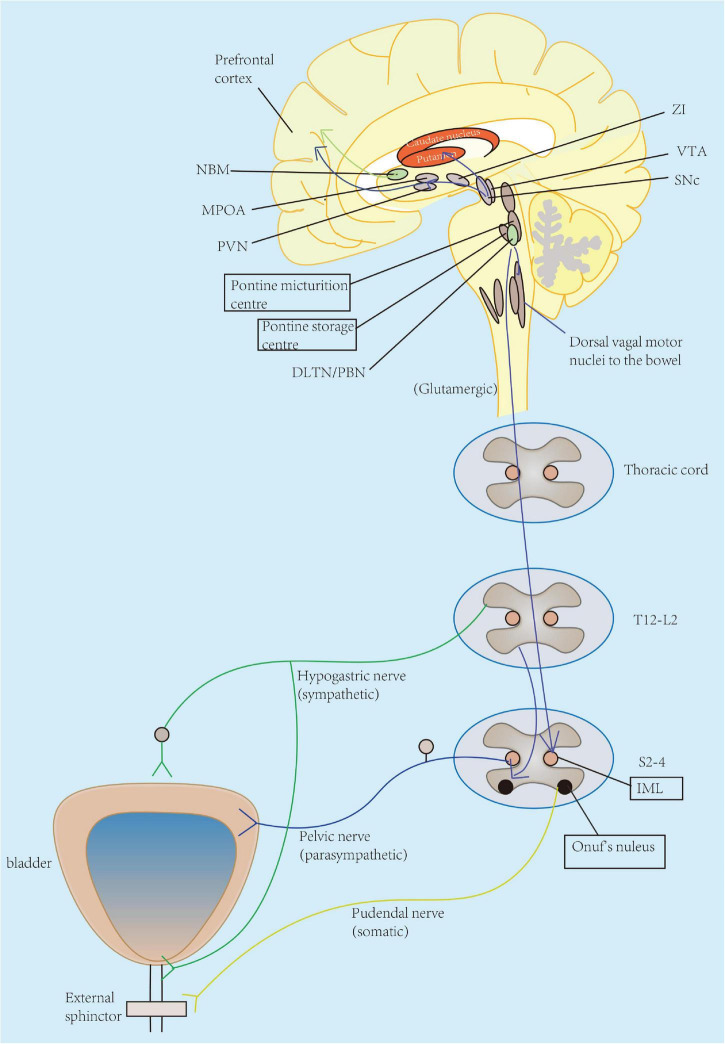
Pathophysiology of OAB symptoms in neurological diseases appears to be multifactorial across all levels of neural control of micturition, from cerebral cortex, brain stem, spinal cord, to the peripheral nerves. The lower urinary tract consists of two major components: the bladder and the urethra. The bladder receives innervation from the parasympathetic pelvic nerve. Parasympathetic (pelvic) nerves could excite the bladder (primarily through activation of muscarinic-3 receptors) and relax the urethra, while sympathetic (hypogastric) nerve enables to inhibit the bladder body (primarily through β-3 adrenergic receptors) and excite the urethra. Pudendal nerve originates from S2 to S4 motor neurons in Onuf’s nucleus and allows to the excitation of the external urethral sphincter. Particularly, urinary storage functions are primarily maintained by the spinal cord reflex with enhanced activity of hypogastric and pudendal nerves innervating the urethra. This function is also under-controlled by the pontine storage center located closely to the pontine micturition center (PMC), hypothalamus, cerebellum, basal ganglia, and frontal cortex. The prefrontal cortex is regarded as the center of planning of complex cognitive behaviors. Other regions also serve as active participants for awareness of visceral sensations, such as the periaqueductal gray (PAG), insula, and anterior cingulate gyrus. ZI, zona incerta; VTA, ventral tegmental area; SNc, substantia nigra pars compacta; NBM, nucleus basalis of Meynert; MPOA, medial preoptic area; PVN, paraventricular nucleus; DLTN, dorsolateral tegmental nucleus; PBN, parabrachial nucleus; IML, intermediolateral cell column; L, lumbar; S, sacral; T, thoracic.

**FIGURE 2:**
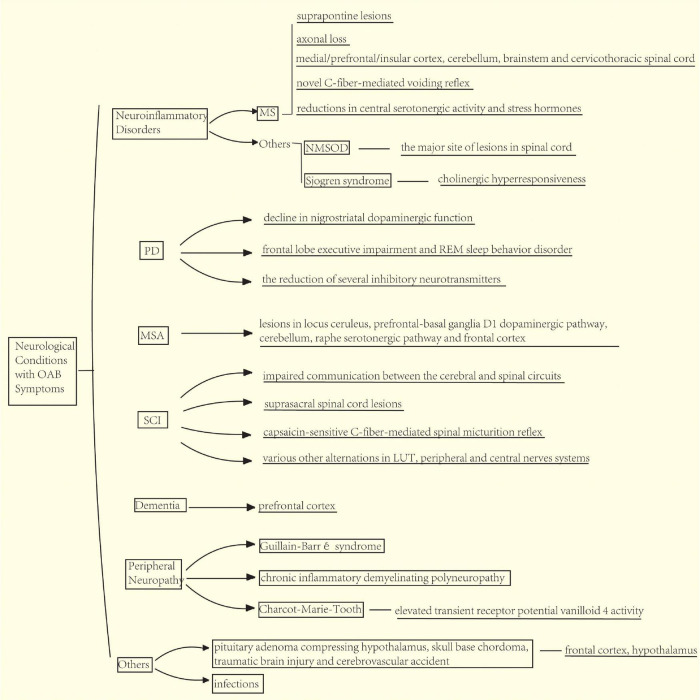
Mechanism of overactive bladder (OAB) symptoms caused by various nervous system diseases. Multiple sclerosis (MS) cause detrusor overactivity (DO) due to the suprapontine lesions, axonal loss, novel C-fiber-mediated voiding reflex, and reductions in central serotonergic activity and stress hormones. Neuromyelitis optica spectrum disorder (NMOSD) causes DO due to spinal cord injury (SCI). Parkinson’s disease (PD) causes DO due to the decline in nigrostriatal dopaminergic function, frontal lobe executive impairment and REM sleep behavior disorder, and the reduction of several inhibitory neurotransmitters in the brain. Multiple system atrophy (MSA) causes lesions in locus coeruleus, prefrontal-basal ganglia D1 dopaminergic pathway, cerebellum, raphe serotonergic pathway, and frontal cortex. SCI leads to DO due to the impaired communication between the cerebral and spinal circuits that coordinate bladder and urethra activities, suprasacral spinal cord lesions, and emergence of a capsaicin-sensitive C-fiber-mediated spinal micturition reflex caused by a reorganization of synaptic connections in the spinal cord. Peripheral neuropathy can also lead to DO. Dementia affecting the prefrontal cortex might also lead to altered central micturition circuit. Other nervous system diseases such as cerebral palsy, pituitary adenoma compressing the hypothalamus, skull base chordoma, traumatic brain injury, and cerebrovascular accident may. Both frontal cortex and hypothalamus are involved.

The aim of this review was to provide a comprehensive and state-of-the-art overview of the close correlation between OAB symptoms and neurological diseases. The summary from over 2,000 published papers showed the complexity and diversity of neurological diseases related to OAB symptoms (Table 1). This study was largely focused on the potential mechanisms underlying the cause of neurogenic OAB symptoms (Figures 1, 2). We aimed to increase the awareness of urological complaints in neurological diseases, inspire robust suitable therapies, and improve the quality of life.

**TABLE 1:** Related neurological diseases associated with overactive bladder symptoms.

Number	Classification	Related diseases
1	Multiple sclerosis and related neuroinflammatory disorders	Multiple sclerosis, acute disseminated encephalomyelitis, neuromyelitis optica spectrum disorder, Sjögren syndrome
2	Parkinson’s diseases	Parkinson’s disease
3	Multiple system atrophy	Multiple system atrophy
4	Spinal cord injury	Spinal cord injury, cauda equina lesions, myelomeningocele, spinal bifida, spinal cord ischemia, spinal stenosis
5	Dementia	Alzheimer’s disease, dementia with Lewy bodies, White matter disease
6	Peripheral neuropathy	Guillain-Barré syndrome, chronic inflammatory demyelinating polyneuropathy, Charcot-Marie-Tooth may
7	Others	Cerebral palsy, cerebrovascular accident, pituitary adenoma compressing the hypothalamus, skull base chordoma, traumatic brain injury, HTLV-1-associated myelopathy/tropical spastic paraparesis, Machado-Joseph disease

*HTLV-1, human T-lymphotropic virus type 1.*

## Methods

A comprehensive electronic literature search was conducted using the PubMed database to identify publications related to the neurological diseases with OAB symptoms. The keywords included the following terms: “overactive bladder,” “nervous system,” “detrusor overactivity,” “detrusor instability,” “unstable bladder,” and “etiology,” used either alone or in combination. The search was restricted to studies published between January 1990 and December 2020. The title and abstract of each article were reviewed for their appropriateness and relevance to the symptoms of OAB in diseases of nervous system. Relevant articles published in English were fully reviewed subsequently.

## Common Basis in Pathophysiology

The pathophysiology of OAB symptoms in neurological diseases appears to be multifactorial across all levels of neural control of micturition, from cerebral cortex, brain stem, spinal cord, to the peripheral nerves. The site and nature of the neurological lesions may affect the appearing time, progression, and severity of OAB symptoms. A better knowledge of the neural component of normal micturition appears to be necessary to the role of neurological diseases in the etiology of OAB symptoms.

In human body, the coordinated activity of the urinary bladder and its outlet is controlled by a complex neural network distributed across parasympathetic, sympathetic, and somatic pathways. Several literature reviews have demonstrated this neural control of micturition reflex (de Groat et al., 2015; Griffiths, 2015; Rahnama’i, 2019). Briefly, parasympathetic (pelvic) nerves could excite the bladder (primarily through activation of muscarinic-3 receptors) and relax the urethra, while sympathetic (hypogastric) nerve enables to inhibit the bladder body (primarily through β-3 adrenergic receptors) and excite the urethra. Pudendal nerve originates from S2 to S4 motor neurons in Onuf’s nucleus and allows to the excitation of external urethral sphincter. Particularly, urinary storage function is primarily maintained by the spinal cord reflex with enhanced activity of hypogastric and pudendal nerves innervating the urethra. This function is also controlled by the pontine storage center located closely to the pontine micturition center (PMC), hypothalamus, cerebellum, basal ganglia, and frontal cortex. PMC is thought to initiate the micturition cycle and receive afferent input from the lumbosacral spinal cord due to bladder distention as well as the prefrontal cortex, which gives social acceptability of voiding (de Groat et al., 2015). The prefrontal cortex is regarded as the center of planning of complex cognitive behaviors. Other regions also serve as active participants for awareness of visceral sensations, such as the periaqueductal gray (PAG), insula, and anterior cingulate gyrus. Furthermore, PAG is considered to mediate switching from storage to voiding, possibly regulated by higher brain regions such as the hypothalamus and prefrontal cortex (Kakizaki et al., 2011). Therefore, any lesion of the central or peripheral nervous system could possibly disrupt the voluntary control of micturition, resulting subsequent in the occurrence of voiding urgency, frequency, incontinence, and nocturia (namely, OAB symptoms).

The OAB symptoms among these neurogenic patients possibly derive from neurogenic detrusor overactivity (DO), which is characterized by involuntary detrusor contractions during bladder filling (Abrams et al., 2003; Defreitas et al., 2003; Karsenty et al., 2008). Although not synonymous of OAB, DO is traditionally thought to be the major causes of urinary urgency/frequency and incontinence (Abrams et al., 2002). Several potential OAB phenotypes have been identified according to urodynamic demonstration of DO, and multiple neurogenic factors could contribute to the development of DO *via* different pathophysiological mechanisms (Peyronnet et al., 2019). First, classic neurogenic DO is thought to be resulting from the loss of supraspinal inhibition control on the micturition reflex or the decreased capacity to functionally integrate afferent information due to the brain damages such as MS, stroke, and PD (Peyronnet et al., 2019). Other increasing evidence supports the idea that deep white matter disease (WMD), mostly in the prefrontal area of the brain, might be the anatomical substrate for brain-related DO (Sakakibara et al., 1999; Griffiths and Tadic, 2008; Tadic et al., 2012). Behavioral therapies (Griffiths et al., 2015), sacral neuromodulation (SNM) (Blok et al., 2006), and posterior tibial nerve stimulation (PTNS) (Finazzi-Agrò et al., 2009) seem appropriate to treat OAB symptoms due to supraspinal lesions.

Second, spinal cord damages such as MS and SCI could induce the occurrence of primitive spinal bladder reflexes mediated by C-fibers afferents, leading to the development of subsequent DO (de Groat, 1997). The C-fibers can be recruited under neuropathic conditions to form a new functional afferent pathway, which can be suppressed by certain drugs such as resiniferatoxin and capsaicin. These findings promote the application of intravesical administration of capsaicin for the treatment of DO in patients with SCI (Chancellor and de Groat, 1999). Of note, spinal lesions below the PMC and above the sacral cord could interrupt spinobulbar pathway and bring about detrusor external sphincter dyssynergia (DESD), such as simultaneous contractions of detrusor and the urethral and/or periurethral striated muscle (Suzuki Bellucci et al., 2012). Voiding features due to spinal cord damages comprise neurogenic DO, DESD, and different types of urinary incontinence.

Third, a growing body of evidence has indicated the coexistence of DO and detrusor underactivity (DU) (Peyronnet et al., 2019; Mancini et al., 2020). Urgency was considered the most common symptom in patients with urodynamically identified DU (Uren et al., 2017). Both DO and DU appear to, at least in part, share the common basis under neurogenic conditions, such as PD, MS, myelitis, and peripheral neuropathy (Balzarro et al., 2019; Mancini et al., 2020). Both DO and DU may be attributed to the impairment of afferents signaling function, central nerve control mechanism, or efferent innervation (Aldamanhori et al., 2018). Currently, no clinically effective drug treatments have been reported for restoring detrusor contractility. Clean intermittent self-catheterization and sacral nerve stimulation (SNM) seem to be helpful to those patients with DO and DU (Gani and Hennessey, 2017).

Fourth, the urethra is likely to play a key role in sensation and continuance, and the activation of urethral afferent signaling system could modulate the micturition reflex, the so-called urethral-vesical reflex (Shafik et al., 2003). Urethrogenic factors, such as the deterioration of urethral tone, are postulated to induce OAB symptoms (Peyronnet et al., 2019). Similarly, a lack of pudendal or central neurological control may also lead to urethral sphincter instability and subsequent urethra-driven OAB (Kirschner-Hermanns et al., 2016). Duloxetine and SNM are likely to be potential treatment options for urethra-driven OAB (Groenendijk et al., 2007; Steers et al., 2007).

Fifth, sensitization of the afferent nerve fibers by the urothelium/suburothelium is implicated in the pathophysiology of neurogenic DO. P2X purinoceptor 3 (P2 × 3) receptor is a type of sensory receptors, and the number of P2 × 3 immunoreactive nerve fibers has been reported an increase in the suburothelium of patients with neurogenic DO (Brady et al., 2004). The expression of other type of sensory receptor, e.g., transient receptor potential vanilloid type 1, also increased in neurogenic patients with DO (Apostolidis et al., 2005).

Lastly, neurogenic-myogenic mechanisms may contribute to the development of neurogenic DO. An early study has demonstrated that partial denervation is attributable to the alternation in smooth muscle properties, leading to enhanced excitability, coordinated myogenic contractions, and enlarged bladder pressure (Turner and Brading, 1997). Due to the complexity of the neural control of micturition, OAB symptoms and DO can be seen as a result of a variety of neurological disorders, including MS, PD, SCI, dementia, and other neurological diseases.

## Animal Models of Neurogenic Overactive Bladder

Overactive Bladder (OAB) is a symptom-based diagnosis in which urgency is the key symptom. The subjective nature of urgency hampers the development of animal models for OAB. Neurological animal models are not directly related to OAB, but they enable to provide a platform for seeking the mechanism of OAB and for assessing novel therapeutic options. Given the high prevalence of OAB symptoms among neurological diseases, a broad spectrum of neurological animal models has been applied to study the OAB symptoms and other lower urinary tract symptoms (LUTS). The commonly used neurological animal models mainly include suprapontine models, spinal cord transection/injury models, and experimental autoimmune encephalomyelitis model.

Suprapontine models are conducted to assess the voiding dysfunctions caused by various central nervous system (CNS) disorders such as PD, dementia, and cerebrovascular events. For instance, several available PD animal models are roughly divided into two groups, namely, toxin-based and genetic models (Kitta et al., 2020). Parkinsonism can be induced by administering the neurotoxins such as 1-methyl-4-phenyl-1,2,3,6-tetrahydropyridine (MPTP) and 6-hydroxydopamine (6-OHDA), which are selective for the rapid degeneration of the nigrostriatal dopaminergic neurons (Schapira et al., 1989; Simola et al., 2007). Other transgenic PD models are developed, including the SNCA (α-synuclein) transgenic models (Matsuoka et al., 2001), DJ-1 KO models (Park et al., 2005), PINK1 KO models (Kitada et al., 2009), and LRRK2 models (Lee et al., 2010). Cerebral infarction animal models could be produced by occlusion of the middle cerebral artery (Yokoyama et al., 1998) or by the induction of midbrain ischemia (Yotsuyanagi et al., 2006), exhibiting voiding dysfunction including bladder overactivity.

Spinal cord transection/injury models are commonly utilized to study DO, DESD, and other types of lower urinary tract (LUT) dysfunction after any injury to the spinal cord, just as traumatic, developmental, infectious, vascular, degenerative injuries, and so on (Ethans et al., 2014; Panicker, 2020). Typically, the SCI model could be achieved by complete transection at different levels of spinal cord in different animals, particularly cats (de Groat et al., 1990) and rats (Shaker et al., 2003; Behr-Roussel et al., 2011). Besides, fetal rats with retinoic acid-induced myelomeningocele could be used to model spina bifida for bladder dysfunction investigation (Danzer et al., 2005).

Experimental autoimmune encephalomyelitis model is capable of mimicking MS-produced bladder dysfunction such as DO. This model can be induced by the activation of immunization with CNS immunogenic compounds, or transfer of encephalogenic T-cell lines from the affected animals (Petry et al., 2000) or infection with Semliki Forest virus (Moss et al., 1989) or coronavirus (McMillan et al., 2014).

## Neurological Conditions With Overactive Bladder Symptoms

### Multiple Sclerosis and Related Neuroinflammatory Disorders

Multiple sclerosis is described as an immune-mediated neuroinflammatory and neurodegenerative disease of the CNS with heterogeneous clinical presentations. According to previous reports, MS is the leading non-traumatic neurological cause of disability in young and middle-aged people in the developed world (Altmann, 2005; Çetinel et al., 2013). Bladder dysfunction is commonly seen in MS, affecting 80–100% of patients during the course of the disease (Minardi and Muzzonigro, 2005). Among them, OAB symptoms are the most common ones, reported by 60–80% of patients with MS. Moreover, a recent study has revealed that the OAB symptoms in MS could highly reach up to 96% (Declemy et al., 2021). Therefore, OAB symptoms remain a considerable clinical challenge to treat.

The mechanism underlying MS-related OAB symptoms is mainly due to the DO induced by suprapontine lesions, with disruption or lack of descending inhibitory impulses from the brain to the spinal cord (Ethans et al., 2014). Many studies about urodynamics in MS cases have demonstrated detrusor hyperreflexia or hyporeflexia (Akkoç et al., 2016; Wang et al., 2016). The primary target of the immune cells in MS is the myelin-producing oligodendrocytes of the CNS, characterized by demyelinated plaques on the brain, brain stem, cerebellum, and/or spinal cord. The myelinated nerve tracts innervating the LUT function would be eventually affected by these demyelinated lesions. Recently, a new concept has emerged that axonal loss, rather than demyelination, is the cause of progressive neurological deficits and much correlated with clinical disability (Dutta and Trapp, 2007). Neuroimaging and pathology studies have proved that in MS, the commonly affected regions relevant to micturition are the medial/prefrontal/insular cortex (Charil et al., 2003), cerebellum (Charil et al., 2003), brain stem (midbrain, Pozzilli et al., 1992; Charil et al., 2003, and pons, Charil et al., 2003; Weissbart et al., 2017), and cervicothoracic spinal cord (Weissbart et al., 2017). Moreover, the predominant cause of DO is thought to be from brain lesions. DO may also be induced by a novel C-fiber-mediated voiding reflex after spinal cord lesions in MS (Sakakibara, 2019). In addition, the reduction in central serotonergic activity and stress hormones in patients with MS may contribute to the occurrence of OAB symptoms (Koutsis et al., 2016).

Other neuroinflammatory disorders of CNS, such as neuromyelitis optica spectrum disorder (NMOSD) (Sakakibara, 2019), acute disseminated encephalomyelitis (Panicker et al., 2009), and Sjögren syndrome (Tarhan, 2013; Lee et al., 2019), can also lead to OAB symptoms. NMOSD is regarded as a type of neuroinflammatory disorders distinct from MS with respect to immunopathogenesis and suitable treatment. NMOSD usually causes more severe longitudinal myelitis or transverse myelitis than MS. Due to the major site of lesions in the spinal cord, NMOSD could cause disturbance of controllable voiding and lead to neurogenic LUTS. De Carvalho and colleagues reported that DO, DESD, and combination of DO and DESD could be urodynamically proven in 20.0%, 23.3%, and 36.6% of patients with NMOSD, respectively (de Carvalho et al., 2016). In Sjögren syndrome, the autoantibodies binding to the M3 muscarinic receptor could result in exocrine dysfunction or cholinergic hyperresponsiveness, subsequently leading to bladder detrusor, smooth muscles contraction, and OAB symptoms (Wang et al., 2004).

### Parkinson’s Diseases

Parkinson’s disease is one of the major diseases characterized pathologically by abnormal α-synuclein aggregation (Ogawa et al., 2017). Lower urinary tract symptoms are one of the main non-motor features in PD, which could be presented during the course of the disease (Defreitas et al., 2003; Winge et al., 2005; Winge and Nielsen, 2012). The epidemiologic data about LUTS in PD are widely scattered. It was recently reported that LUTS occurred in 27–63.9% of patients with PD and increased with the severity of PD (Barone et al., 2009; Ogawa et al., 2017). Another previous report estimated that highly up to 80% of patients with PD may suffer from LUTS (McDonald et al., 2017). Overactive bladder symptoms are the most common type of LUTS (Shah and Weiss, 2012; McDonald et al., 2017) and could be as early symptoms than motor-related ones among patients with PD (Roy et al., 2020). Overactive bladder symptoms can be treated as a warning of progression to PD dementia (Xu et al., 2019). Additionally, patients with PD frequently have LUTS, such as nocturia, increased urinary frequency, and urinary incontinence, overlapping with those of OAB symptoms (Ogawa et al., 2017). During urodynamic testing, about 36–93% of patients with PD showed uninhibited contractions or DO (Ogawa et al., 2017).

In patients with PD, the etiology of OAB symptoms is centrally mediated and modulated in complex ways that are not fully understood. Strong evidence indicated that a decline in nigrostriatal dopaminergic function plays a crucial role in this progress (Sakakibara et al., 2001; Winge et al., 2005). Overactive bladder symptoms may arise from the disruption of the complex control loops within the context of neurodegeneration, instead of a focal lesion (Winge and Fowler, 2006; Campeau et al., 2011). For instance, basal ganglia could interfere with the function of the PMC. In PD, altered signaling in the nigrostriatal dopaminergic system results in a partial or total disconnection of the micturition reflex from voluntary control and subsequent uninhibited bladder contractions (Blackett et al., 2009). Besides, several studies confirmed and expanded the views above. Functional neuroimaging studies revealed a significant decline of dopamine transporter imaging in the brain of patients with PD with LUTS (Sakakibara et al., 2001; Winge et al., 2005). In animal studies, the disruption of nigrostriatal dopaminergic system was also proved to produced DO (Albanese et al., 1988; Yamamoto et al., 2005a), which could be inhibited by stimulating D1-like dopamine receptor with agonists or pergolide (Yoshimura et al., 2003). Additionally, enhanced activity of the adenosine A_2A_ system in the brain may contribute to DO in PD, which could be suppressed by A_2A_ antagonist ZM 241385 (Kitta et al., 2012). Clinical application of A_2A_ receptor antagonists such as istradefylline may also be a promising candidate for the treatment of LUTS in patients with PD (Kitta et al., 2016). Moreover, several specific non-motor symptoms are also known to be correlated with LTUS in PD (Sakakibara et al., 2013; Rana et al., 2015). A cross-sectional study suggested that frontal lobe executive impairment and rapid eye movement (REM) sleep behavior disorder accompanied with a higher prevalence of OAB symptoms in patients with PD, possibly due to the overlap of locus coeruleus and pontine nucleus with some regions that control micturition (Xu et al., 2019). Other postulated mechanisms underlying the relationship between OAB symptoms and PD are the reduction of several inhibitory neurotransmitters in the brain related to micturition, such as γ-aminobutyric acid, serotonin, and norepinephrine (Boeve et al., 2007; Buddhala et al., 2015).

### Multiple System Atrophy

Multiple system atrophy (MSA) is a rare type of neurodegenerative disorders characterized by varied combinations of autonomic (orthostatic or bladder) with motor (parkinsonian or cerebellar dysfunction; Tsuchiya et al., 2020). Patients with MSA could present Parkinson-like motor symptoms and some similar LUTS. MSA may initially present with bladder dysfunction, particularly urinary retention. Over 90% of patients with MSA could have LUTS, which are more prevalent and severe than those with PD (Sakakibara et al., 2000; Yamamoto et al., 2011). About 50% of patients with MSA could also develop OAB symptoms (Roy et al., 2020). Moreover, DO could be confirmed in 33–100% patients with MSA during urodynamic investigations (Ogawa et al., 2017). The occurrence of OAB symptoms and other LUTS may arise from lesions in the area relevant to micturition, including locus coeruleus, prefrontal-basal ganglia D1 dopaminergic pathway, cerebellum, raphe serotonergic pathway, and frontal cortex (Gilman et al., 2008; Cykowski et al., 2015). Tsuchiya et al. (2020) also elucidated that DO occurred independently from motor disorder in MSA.

### Spinal Cord Injury

Spinal cord injury (SCI) arises from traumatic and non-traumatic events, with an annual incidence of up to 40 cases per million people. The prevalence of LUTS ranges from 20 to 88.3% in patients with traumatic SCI and 5.9% to 90% in patients with non-traumatic SCI (Ruffion et al., 2013; Fergany et al., 2017). The most common finding during urodynamic studies is DO in patients with SCI, with a prevalence ranging from 11 to 85% (Ruffion et al., 2013). Detrusor overactivity usually emerges when spinal reflexes return.

The appearance of LUTS, including OAB symptoms, in patients with SCI possibly derive from the impaired communication between the cerebral and spinal circuits that coordinate bladder and urethra activities (de Groat et al., 2015). The degree of urinary symptoms is related to the disease process itself, site of affected, and severity of neurological impairment. Suprapontine or suprasacral spinal cord lesions could induce storage dysfunctions, leading to DO. Lesions in the spinal cord may cause DESD and incomplete bladder emptying. Sacral or infrasacral lesions result in denervation of bladder and/or sphincters with incompetent sphincter and poorly sustained/absent detrusor contractions (Vichayanrat et al., 2021). As aforementioned, DO may be correlated with the emergence of a capsaicin-sensitive C-fiber-mediated spinal micturition reflex caused by a reorganization of synaptic connections in the spinal cord (Banakhar et al., 2012). As shown in chronic spinalized cats, administration of capsaicin could desensitize TRPV1-expressing C-fiber afferent pathways and completely block DO (de Groat et al., 1990; Cheng et al., 1999). Similarly, desensitization of C-fiber afferents by capsaicin pretreatment could also inhibit DO and DESD in chronic SCI rats (Cheng et al., 1995; Seki et al., 2004). In addition, cold-sensitive C-fiber afferents likely promote the occurrence of SCI-related DO and DESD mediated by transient receptor potential melastatin 8 (TRPM8) (Fall et al., 1990).

The hyperexcitability of C-fiber afferent pathway may be attributed to a wide variety of peripheral-to-central mechanisms. For instance, transient receptor potential ankyrin 1 (TRPA1) in the suburothelial nerve fibers may play a role in the C-fiber-mediated DO in SCI (Andrade et al., 2012). Increased activation of P2 × 2/3 receptors in the bladder is shown to stimulate bladder afferents and induce DO in rats following SCI (Smith et al., 2008; Munoz et al., 2012). After SCI, bladder afferent neurons seem to be more sensitive to the bladder stimuli due to pathophysiological alternations, such as ion channel transformations (Yoshimura and de Groat, 1997; Takahashi et al., 2013). Other changes in lumbosacral spinal cord, including increased expression of pituitary adenylate cyclase-activating polypeptide, could also contribute to the emergence of DO in SCI (Zvarova et al., 2005).

In addition, various other alternations in LUT, peripheral nerve system, and CNS also contribute to the development of DO after SCI, such as aberrant expressions/activation of M2 muscarinic receptor (Pontari et al., 2004), neurotrophic factors (Lamb et al., 2004), glutamate system (Yoshiyama et al., 1999), glycine (Miyazato et al., 2003), and γ-aminobutyric acid (Miyazato et al., 2008).

Overactive bladder (OAB) symptoms can also occur in other spinal cord diseases, including spina bifida (Dorsher and McIntosh, 2012), cauda equina lesions (Deffontaines Rufin et al., 2010), myelomeningocele (Dean and Long, 2011), spinal cord ischemia (Grosse et al., 2005), and spinal stenosis (Semins and Chancellor, 2004).

### Dementia

Dementia diseases, such as Alzheimer’s disease (AD), dementia with Lewy bodies (DLB), and subacute combined degeneration, are also known as independent-risk factors for OAB symptoms (Takahashi et al., 2012). Previous studies reported a much higher prevalence of OAB in patients with AD aged 56–92 years (72.6%) than that in the general population, and almost twice as high as that in the general population above 75 years of age (Jung et al., 2017). Dementia with Lewy bodies is the second most common degenerative cause of dementia and OAB symptoms are more prevalent in DLB than in AD and PD (Ransmayr et al., 2008). Detrusor overactivity on urodynamic studies could be identified in 71.4–92% patients with DLB (Sakakibara et al., 2005; Ransmayr et al., 2008; Tateno et al., 2015). White matter disease is a chronic, bilateral form of cerebrovascular disease, leading to a high prevalence of OAB (up to 90%) (Sakakibara et al., 2014). The pathological mechanisms for OAB symptoms in dementia diseases remain unclear, but experimental and neuroimaging studies have suggested that the prefrontal cortex is critical for the higher control of voiding and enhanced bladder sensation might also result from altered central micturition circuit (Tsunoyama et al., 2011).

### Peripheral Neuropathy

Peripheral neuropathy refers to a broad range of disorders causing damage and dysfunction of the nerves of the peripheral nervous system (Barrell and Smith, 2019). The damage to the peripheral nerves related to micturition reflex could bring about various types of LUTS including OAB symptoms. For example, about 75% of patients with Guillain-Barré syndrome developed micturition problems and both DO and DU were commonly seen in urodynamic analysis (Sakakibara et al., 2009). Patients with chronic inflammatory demyelinating polyneuropathy (CIDP) reported less LUTS in comparison to Guillain-Barré syndrome and the rate of LUTS ranged from 2 to 25%. Voiding difficulty and urinary urgency are the major urinary symptoms in CIDP. Charcot-Marie-Tooth (CMT), a type of hereditary peripheral neuropathy, may also occur with OAB symptoms, possibly due to the elevated transient receptor potential vanilloid 4 activity (Nilius and Owsianik, 2010). Thus, LUTS are usually rare in patients with CMT and CIDP. A careful exclusion of urological comorbidities and other neurological conditions, such as stroke and spinal stenosis, is required in patients with CMT and CIDP who present with LUTS.

### Other Neurological Diseases

Other brain lesions are also relevant to OAB symptoms, including cerebral palsy, pituitary adenoma compressing the hypothalamus (Yamamoto et al., 2005b), skull base chordoma (Akhavan-Sigari et al., 2014), traumatic brain injury (Sakakibara, 2015), and cerebrovascular accident (Gupta et al., 2009). The incidence of LUTS in these patients ranges from 14 to 53%, mostly OAB symptoms, and is much higher when the frontal cortex is involved (Sakakibara, 2015). As mentioned above, supraspinal lesions can lead to neurogenic OAB symptoms due to the loss of inhibition control on the micturition reflex or the decreased capacity to functionally integrate afferent information. Besides, hypothalamic lesions can lead to severe LUT dysfunction in both the storage and voiding phases of micturition, suggesting the crucial role of the hypothalamus in regulating micturition in humans (Yamamoto et al., 2005b).

Additionally, OAB symptoms could occur in patients with infections in the nervous system, including meningitis-retention syndrome (Tateno et al., 2011), human T-lymphotropic virus type 1-associated myelopathy/tropical spastic paraparesis (Santos et al., 2012; Rahnama’i, 2019), and human immunodeficiency virus (HIV)-associated neuropathy (Hermieu et al., 1996). Individuals with Machado-Joseph disease (Musegante et al., 2011) could also report OAB symptoms.

## Conclusion

The etiology of OAB is multifactorial and may arise from a broad spectrum of medical conditions, in particular neurogenic ones. This study provided a comprehensive overview of OAB-related neurogenic disorders and summarized the compensatory mechanisms underlying the emergence of OAB symptoms in these neurogenic disorders. This may provide a robust rationale for therapy, as it could tackle several mechanisms increasing the chance of therapeutic success. However, given limited publications could present neurogenic OAB symptoms totally and clearly; further studies including etiology, epidemiology, and treatment are required.

## Author Contributions

YG conceived the idea for the manuscript. CQ drafted the manuscript with significant contribution from YG. YW revised the manuscript. All authors contributed to the article and approved the submitted version.

## Conflict of Interest

The authors declare that the research was conducted in the absence of any commercial or financial relationships that could be construed as a potential conflict of interest.

## Publisher’s Note

All claims expressed in this article are solely those of the authors and do not necessarily represent those of their affiliated organizations, or those of the publisher, the editors and the reviewers. Any product that may be evaluated in this article, or claim that may be made by its manufacturer, is not guaranteed or endorsed by the publisher.
